# Water Resistance and Creep Behavior of Heat-Treated Moso Bamboo Determined by the Stepped Isostress Method

**DOI:** 10.3390/polym13081264

**Published:** 2021-04-13

**Authors:** Teng-Chun Yang, Tung-Lin Wu, Chin-Hao Yeh

**Affiliations:** 1 Department of Forestry, National Chung Hsing University, Taichung 402, Taiwan; harrison19960219@gmail.com; 2 Department of Wood Science and Design, National Pingtung University of Science and Technology, Pingtung 912, Taiwan; tonywu@mail.npust.edu.tw

**Keywords:** moso bamboo, heat treatment, water resistance, creep behavior, stepped isostress method (SSM)

## Abstract

The influence of heat treatment on the physico-mechanical properties, water resistance, and creep behavior of moso bamboo (*Phyllostachys pubescens*) was determined in this study. The results revealed that the density, moisture content, and flexural properties showed negative relationships with the heat treatment temperature, while an improvement in the dimensional stability (anti-swelling efficiency and anti-water absorption efficiency) of heat-treated samples was observed during water absorption tests. Additionally, the creep master curves of the untreated and heat-treated samples were successfully constructed using the stepped isostress method (SSM) at a series of elevated stresses. Furthermore, the SSM-predicted creep compliance curves fit well with the 90-day full-scale experimental data. When the heat treatment temperature increased to 180 °C, the degradation ratio of the creep resistance (*r*_d_) significantly increased over all periods. However, the *r*_d_ of the tested bamboo decreased as the heat treatment temperature increased up to 220 °C.

## 1. Introduction

Bamboo has been of interest in academia and industry as a natural resource due to its fast growth rate compared to other plants and because it is an eco-friendly and renewable material. Recently, bamboo-based products, such as reconstituted densified bamboo boards [1,2,3], laminated bamboo lumber [4,5], and bamboo fiber-reinforced polymer composites [6,7], have been increasingly developed in various regions. However, bamboo has a high hygroscopicity and low biological durability since it is a lignocellulosic material. To resolve these drawbacks, heat treatment, which is a physical and eco-friendly modification, has been considered in the bamboo-based product industry. Several previous studies have treated the bamboo under various temperatures, durations, and media (air, nitrogen, oil, and steam) [8,9,10,11]. The findings of these studies indicated that heat treatment decreases the hygroscopicity of bamboo and improves its dimensional stability, but it reduces the mechanical properties. Yang et al. [8] reported that the moisture content, density, and flexural properties decreased, and moisture-excluding efficiencies and anti-shrinking of bamboo improved as the treatment temperature increased. Zhang et al. [9] stated that the contents of the chemical components (holocellulose and *α*-cellulose) and the modulus of rupture (MOR) reduced with the increase in temperature and duration when bamboo was treated above 160 °C. Yang et al. [10] investigated and observed that the bamboo treated at a higher temperature had a high thermal stability and Poisson’s ratio, an increase in cellulose crystallinity, hemicellulose deacetylation, and cross-linking of lignin condensation. Additionally, Marasigan et al. [12] explored that heat treatment caused a change in the surface color and an improvement in the wettability property of two bamboo species in Philippines. Brito et al. [13] affirmed that heat treatment improved biological resistance of bamboo to brown rot fungi, but not to termites. These previous studies mainly focused on physico-mechanical properties and biological durability of the heat-treated bamboo. However, the available information on the creep behaviors of the heat-treated bamboo lacks detail.

The phenomenon of creep is the permanent inelastic deformation of a material caused by a sustained applied load and affects serviceability. Additionally, creep has the ability to change the characteristics of a structural element or system, possibly resulting in structural failure. Therefore, creep behavior is one of the critical issues in the structural design of materials, particularly anisotropic materials. A few previous studies investigated the creep of untreated bamboo [14,15,16]. However, the available information on the creep of heat-treated bamboo lacks detail. Generally, a long-term and full-scale creep test is conducted to estimate the service life of a material, but it is prohibitively expensive and time-consuming. Therefore, an accelerated creep test based on the stepped isostress method (SSM) has been developed to reduce the test time and to predict the full-scale creep of a material. Previous studies [17,18] have reported that the SSM could successfully evaluate the creep behavior of various materials, such as wood and wood-based products. However, there is little information available on the creep properties of heat-treated bamboo samples. Accordingly, the present study aims to investigate the effects of heat treatment on the dimensional stability of moso bamboo after a water absorption test and to determine its creep resistance with the SSM.

## 2. Materials and Methods

### 2.1. Materials and Heat Treatment Process

The middle section of a three-year-old moso bamboo (*Phyllostachys pubescens*) culm was purchased from a local factory in Nan-Tou County, Taiwan. The bamboo culm was split and peeled to obtain bamboo strips with dimensions of 120 mm (L) × 8 mm (*T*) × 5 mm (*R*). The density of all the samples selected was in the range of 700 to 750 kg/m^3^. For heat treatment, the samples were heated at 180 °C and 220 °C for 2 h under nitrogen at atmospheric pressure in a conventional oven (JB-27, ProKao Instrument Co., Taichung, Taiwan). Additionally, the untreated sample and samples heat-treated at 180 °C and 220 °C were denoted as Mo_NT_, Mo_T180_, and Mo_T220_, respectively. Prior to testing, all the samples were conditioned at 25 °C and 65% relative humidity (RH) for 4 weeks.

### 2.2. Physical and Flexural Properties

#### 2.2.1. Density

The density (*ρ*) of a sample before and after heat treatment and the loss rate in the density (*DLR*) were calculated as

*ρ* (kg/m^3^) = *m*/*v* (1)

*DLR* (%) = (*ρ*_N_ − *ρ*_h_)/*ρ*_N_ × 100 (2)

where *m* and *v* are the mass and volume of the sample, respectively, and *ρ*_N_ and *ρ*_h_ are the densities of the sample before and after heat treatment, respectively.

#### 2.2.2. Moisture Content

The moisture content (*MC*) of the sample before and after heat treatment and the loss rate in the moisture content (*MLR*) were determined as

*MC* (%) = (*m*_u_ − *m*_o_)/*m*_o_ × 100 (3)

*MLR* (%) = (*MC*_N_ − *MC*_h_)/*MC*_N_ × 100 (4)

where *m*_u_ and *m*_o_ are the masses of the sample after conditioning and oven-drying, and *MC*_N_ and *MC*_h_ are the moisture content of the sample before and after heat treatment.

#### 2.2.3. Flexural Properties

The samples with an outer layer on the tension side were tested in a three-point flexural test at a span of 84 mm and a crosshead speed of 2 mm/min. The modulus of rupture (MOR), modulus of elasticity (MOE), and strain at ultimate load (*ε*_u_) were assessed with the following equations:

MOR (MPa) = 3*P*_u_*L*/(2*bh*^2^) (5)

MOE (MPa) = *P*_p_*L*^3^/(4*δbh*^3^) (6)

*ε*_u_ (%) = 6*Dh*/*L*^2^ (7)

where *P*_u_ is the ultimate load (N), *L* is the span of the sample (mm), *b* is the width of the sample (mm), *h* is the thickness of the sample (mm), *P*_p_ is the load difference within the proportional limit (N), *δ* is the deflection at the midspan under *P*_p_ (mm), and *D* is the deflection under *P*_u_ (mm).

#### 2.2.4. Water Absorption Test

Before testing, all the samples were previously oven-dried at 105 °C. Afterward, the samples were fully immersed in water at 20 °C for 24 h, and the weight and volume were then measured. According to ASTM D1037-12, the water absorption rate (*WAR*) and volume swelling coefficient (*VSC*) of untreated and heat-treated samples were determined using the following equations:

*WAR* (%) = (*W*_w_ − *W*_o_)/*W*_o_ × 100 (8)

*VSC* (%) = (*V*_w_ − *V*_o_)/*V*_o_ × 100 (9)

where *W*_w_ and *V*_w_ are the weight and volume of the sample after immersion in water, and *W*_o_ and *V*_o_ are the weight and volume of the sample after oven-drying. Furthermore, the anti-swelling efficiency (*ASE*) and anti-water absorption efficiency (*AWAE*) were calculated as

*ASE* (%) = (*VSC*_N_ − *VSC*_h_)/*VSC*_N_ × 100 (10)

*AWAE* (%) = (*WAR*_N_ − *WAR*_h_)/*WAR*_N_ × 100 (11)

where *VSC*_N_ and *WAR*_N_ are the *VSC* and *WAR* of the sample before heat treatment, and *VSC*_h_ and *WAR*_h_ are the *VSC* and *WAR* of the sample after heat treatment.

### 2.3. Creep Test

**Figure 1:**
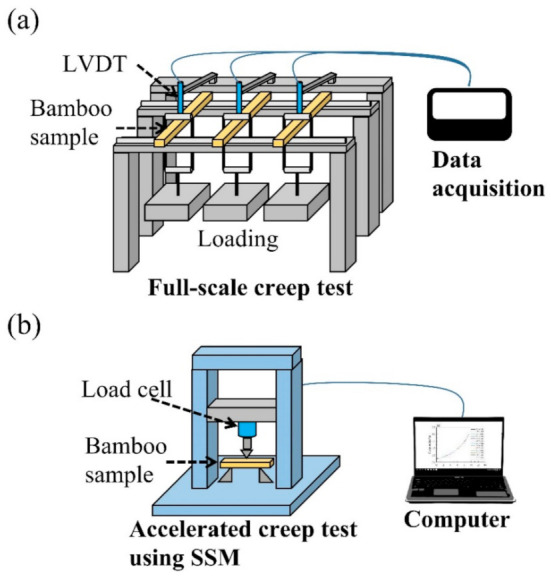
Schematic diagrams of the full-scale creep test (**a**) and accelerated creep test using SSM (**b**).

A conventional experimental creep test was implemented to serve as a basis for comparison with an accelerated creep test using the SSM, as shown in Figure 1a. The sustained stress was 20% of the average breaking load (ABL) for a period of 90 days. The vertical deflection at the midspan was recorded using a linear variable differential transducer (LVDT). Three samples of each bamboo treatment were tested.

The SSM was implemented using a universal testing machine (Shimadzu AG-10kNX, Tokyo, Japan) equipped to predict the long-term and full-scale experimental creep behavior of untreated and heat-treated bamboo samples (Figure 1b). To investigate the SSM creep tests using various testing parameters, a creep test following the SSM was conducted using a series of isostresses from 20% to 80% ABL, with intervals of 5%, 7.5%, and 10% ABL. The reference stress and the dwell time for each isostress were 20% ABL and 2 h, respectively. A master curve, which involves the creep strain at a reference stress and shifting the timescale of the measured creep curves, was constructed with the SSM data and Equation (12):

*ε*(*σ*_r_, *t*) = *ε*(*σ*, *t*/*α*_σ_) (12)

where *ε* is the creep strain as a function of stress and time, *σ*_r_ is the reference stress, *σ* is the elevated stress, and *α*_σ_ is the shift factor. Additionally, the activation volume was calculated to estimate the shift factor (*α*_σ_) based on the Eyring model, which describes the creep rate with the stress level [19,20]:

(13) $$\log\;\alpha_{\sigma }\;=\;\log\left(\frac{\dot{\varepsilon }}{{\dot{\varepsilon }}_{r}}\right)\;=\;\frac{V*}{2.30kT}\left(\sigma -\sigma_{\mathrm{ref}}\right)$$

where $\dot{\varepsilon }$ is the creep rate at the elevated stress (*σ*), ${\dot{\varepsilon }}_{r}$ is the creep rate at the reference stress (*σ*_ref_), V* is the activation volume, *k* is Boltzmann’s constant, and *T* is the absolute temperature. Furthermore, the creep compliance (*J*(*t*)) was calculated to estimate the creep behavior of the samples due to the changes in the flexural strength among all the samples and is given in Equation (14) [21]:

*J*(*t*) = *D*(*t*) (4*bh*^3^/*PL*^3^) (14)

where *D*(*t*) is the time-dependent deflection, *b* is the width of the sample, *h* is the thickness of the sample, *t* is the elapsed time, *P* is the applied load, and *L* is the length of the span. All the samples were held at 25 °C and 65% RH during the accelerated and experimental creep tests.

### 2.4. Analysis of Variance

All of the results are expressed in terms of the mean ± the standard deviation (SD). The significance of the differences was calculated using Scheffe’s test; *p* < 0.05 was considered to be significant.

## 3. Results and Discussion

### 3.1. Physical and Flexural Properties

The physical properties of the studied untreated and heat-treated bamboo samples are shown in Table 1, including the density (*ρ*) and moisture content (*MC*). The density significantly decreased from 721 (MO_NT_) to 642 g/cm^3^ when the heat treatment temperature increased to 220 °C. The results showed that the density loss rate (*DLR*) was 4.0% when the bamboo sample was heated at 180 °C (MO_T180_). This phenomenon is related to the mass loss of the heat-treated sample due to the thermal degradation of chemical components, such as hemicellulose and extractives [3,8,10,22]. Furthermore, the *DLR* value significantly increased to 10.8% with heat treatment at 220 °C (MO_T220_), which indicated that the decrease in the *ρ* value for MO_T220_ was higher than that for MO_T180_. This is attributed to the considerable decomposition of the noncrystalline cellulose and hemicellulose [3,8,10,22]. Additionally, the moisture content (*MC*) significantly decreased from 8.9% (MO_NT_) to 6.0%, and 5.0% for MO_T180_ and MO_T220_, respectively. This result revealed that the *MC* value of the bamboo samples decreased as the heat treatment temperature increased. MO_NT_ exhibited the highest *MC* value among all the samples since the bamboo is sensitive to moisture. However, the loss rate of the *MC* value (*MLR*) was 33.5% for MO_T180_ and 44.9% for MO_T220_. Yang et al. [8] and Salim et al. [23] reported that the *MLR* value of bamboo decreased due to a decreasing hygroscopy with increasing heat treatment temperature. In view of these results, heat treatment could have modified the bamboo sample to become hydrophobic due to the increase in the cellulosic crystallinity and hemicellulose degradation [24,25]. Additionally, the decrease in the hygroscopicity of the heat-treated bamboo is related to the hornification effect, which is the consequence of irreversible hydrogen bonding during heat treatment [26]. The flexural properties of all the samples are presented in Table 1. Mo_NT_ exhibited flexural properties with MOR, MOE, and *ε*_u_ values of 135 MPa, 10.8 GPa, and 2.9%, respectively. MOR and *ε*_u_ significantly decreased by 32.6% and 62.1%, respectively, when the samples were thermally treated at 220 °C. Previous studies have indicated that the mechanical properties of bamboo were reduced at higher treatment temperatures [8,9]. However, there was no significant difference in the MOE among all the samples, which remained in the range of 10.8 to 11.6 GPa. Similar results were reported by Yang et al. [8] and Manalo and Acda [27]. The decline in the mechanical properties of the heat-treated bamboo samples could be related to the depolymerization of hemicellulose and cellulose and the separation of the hemicellulose−lignin copolymer [28,29]. Bhuiyan and Sobue [30] found that the cellulose in wood degrades under heat treatment at 200−240 °C. The results indicated that the flexural properties of the bamboo samples were considerably influenced by the heat treatment.

The *WAR*s and *VSC*s of samples heated at different treatment temperatures after water immersion are listed in Table 2. Mo_NT_ had the highest *WAR* (49.3%) and *VSC* (10.6%) values among all the samples. This is attributed to the fact that bamboo tissue contains vessels and parenchyma cells that enable water penetration, and hydrogen bonds with water molecules are formed due to the many free hydroxyl groups in the bamboo cell wall [31,32]. By contrast, the *WAR* value significantly decreased to 35.2%, while the heat treatment increased to 180 °C and then leveled off at up to 220 °C. In the same way, the *AWAE* values of the heat-treated samples were in the range of 28.3% to 32.4%. The results showed that the *WAR* tended to decrease as the treatment temperature increased to 180 °C. Moreover, the statistical analysis indicated that there were no significant differences among the *WAR* results of the bamboo samples when the treatment temperature was more than 180 °C. Furthermore, the *VSC* values were 7.9% and 4.5% for Mo_T180_ and Mo_T220_, respectively. The *ASE* values increased from 24.1% to 56.3% with increasing treatment temperature from 180 °C to 220 °C. According to these results, large *AWAE* and *ASE* values indicate better performance in reducing the water absorption and volume swelling of the bamboo during the water absorption test. Therefore, these results demonstrated that the bamboo samples heated at higher treatment temperatures had higher water resistances. These findings are mainly ascribed to the hydrolyzation of hemicelluloses during the heat treatment, which decreases the hygroscopicity of bamboo [8,33,34].

### 3.2. Master and Predicted Curves by the SSM

**Figure 2:**
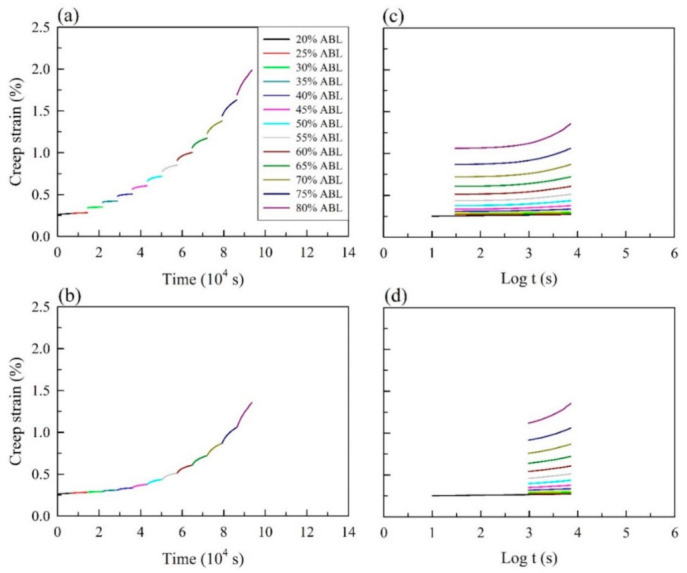
(**a**) The SSM creep strain of the Mo_NT_ (reference stress: 20% ABL; interval stress: 5% ABL; dwell time: 2 h). The processing of the test data of the SSM method for Mo_NT_: (**b**) vertical shifting, (**c**) rescaled creep curves, and (**d**) eliminating the period before the onset time of each stress step.

In this study, accelerated creep tests using the SSM at a range of elevated stresses were performed. As an example, Figure 2a shows the flexural creep strain of Mo_NT_ at a reference stress of 20% ABL with a 5% stepwise increase in the ABL and a dwell time of 2 h over the actual time. The master curve of the SSM was constructed by the following four steps of test data adjustments: (1) vertical shifting, (2) rescaling, (3) eliminating, and (4) horizontal shifting.

**Figure 3:**
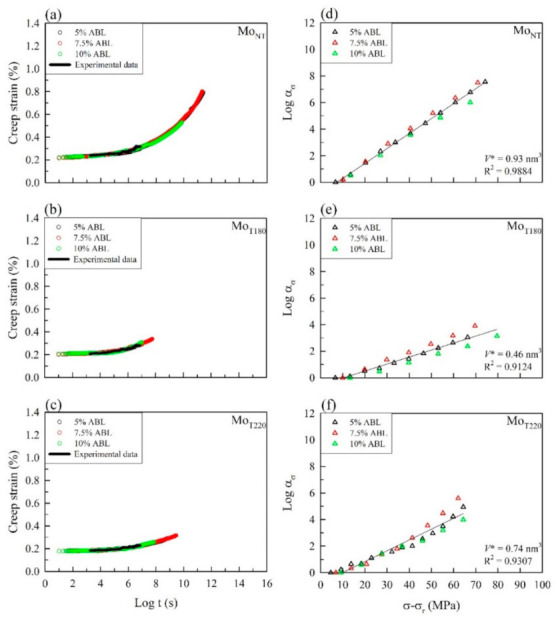
(**a**–**c**) Master curves after horizontal shifting from different SSM testing parameters and experimental data. (**d**–**f**) Relationships between the time-stress shift factor and stress level from different SSM testing parameters.

As shown in Figure 2b, a continuous creep strain curve was generated through vertical shifting to link the start of the current curve to the end of the previous curve at each load step. The immediate strain jumps between the load steps were subtracted by vertical shifting since there was no creep strain at each jump under instantaneous strain. According to a previous study [35], the rescaling and eliminating processes were carried out using a modified method as the first two steps in the SSM. A series of independent creep curves from stepwise sequential stress increases were shifted to the reference stress level (20% ABL) along the log scale of the time axis, as shown in Figure 2c. Additionally, the time before the onset of the primary creep region was influenced by the stress-level history, and it was eliminated from the individual curves (Figure 2d). Subsequently, the individual creep curves were horizontally shifted to construct the master curve along the time axis according to the shift factor log(*α*_σ_). Figure 3a–c illustrates the smooth master curves of all the untreated and heat-treated samples obtained from different testing parameters after the SSM processing steps. Furthermore, all of the creep master curves of the untreated and heat-treated bamboo samples were fitted with each 90 days of experimental creep strain data. The results demonstrated that the creep master curves of the bamboo samples using the SSM procedure were highly consistent with the experimental data, and they were not affected by the test conditions for a given bamboo sample. Relationships between the time-stress shift factor and stress level from different SSM testing parameters are shown in Figure 3d–f. The plot of the shift factor versus the stress level exhibited a nearly straight line with a linear regression coefficient of determination (R^2^) greater than 0.90. Previous studies have reported that the construction of master curves for various materials was not influenced by different SSM testing parameters, indicating a consistent creep mechanism [17,18,19,35]. Based on the Eyring model (Equation (13)), the activation volume (V*) of Mo_NT_ was calculated from the linear slope to 0.93 nm^3^, while the V* values were 0.46 nm^3^ and 0.74 nm^3^ for Mo_T180_ and Mo_T220_, respectively. This result implied that the V* values of the heat-treated samples were lower than those of the untreated sample. The change in activation volume corresponded to chain slippage of crystalline lamellae in the lamellar clusters [36].

**Figure 4:**
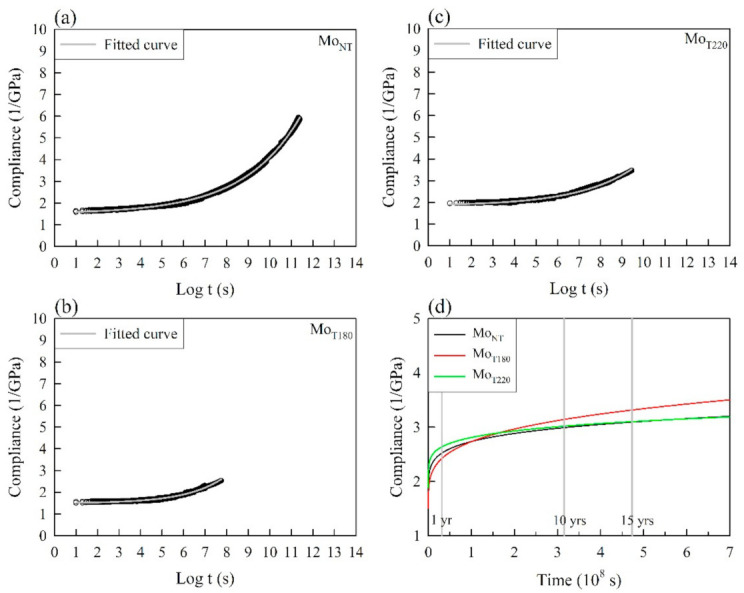
SSM-fitted creep curves of various samples on a log time scale (**a**–**c**) and normal time scale (**d**).

On the other hand, the SSM-predicted compliance master curves of the untreated and heat-treated bamboo samples on a log time scale are shown in Figure 4a–c. An exponential growth equation with three parameters was fitted with the creep master curves of all the samples, which is given by the following equation:

(15) $$S\left(t\right)\;=S_{0}+ae^{bt}$$

where *S*(*t*) is the time-dependent compliance value, *S*_0_ is the instantaneous elastic compliance value, *a* and *b* are constant values, and *t* is the elapsed time. The model fit the master curves very well over the entire period, and the R^2^ values for all the samples were greater than 0.98 (Table 3). Figure 4d presents the SSM-fitted creep curves of all the samples on a normal time scale over 20 years. Accordingly, the *S*_0_ values were 1.51, 1.49, and 1.84 GPa^−1^ for Mo_NT_, Mo_T180_, and Mo_T220_, respectively. When the heat treatment temperature increased to 180 °C, the compliance values significantly increased to 3.14 and 3.32 GPa^−1^ at 10 and 15 years, although the results were different in the first year. However, the Mo_T220_ compliance was similar to that of Mo_NT_ when the sample was thermally treated at 220 °C. In addition, the *b* values of all the heat-treated samples were higher than that of Mo_NT_ (0.38). The results indicated that the heat-treated samples had a high creep rate. Furthermore, the degradation ratio of the creep resistance (*r*_d_) was calculated to estimate the creep behavior of the heat-treated samples compared to that of Mo_NT_ under long-term conditions, which is described by the following equation (Equation (16)):

(16) $$r_{d}\;(\%)\;=\;\left[\frac{{S(t)}_{h}}{{S(t)}_{N}}-1\right]\;\times \;100$$

where *S*(*t*)_N_ and *S*(*t*)_h_ are the time-dependent compliances for the untreated and heat-treated samples, respectively. As listed in Table 3, Mo_T180_ had the highest *r*_d_ value observed in this work, and the corresponding creep resistance increasingly degraded to 5.0% and 7.2% over 10 and 15 years, respectively. Moreover, when the heat treatment temperature increased above 180 °C, *r*_d_ could be reduced to 0.9% at 10 years and 0.3% at 15 years (Mo_T220_). Accordingly, these results demonstrated that the long-term creep resistance of the tested bamboo sample would be degraded by heat treatment.

## 4. Conclusions

The present study investigated the influence of heat treatment temperature on the physico-mechanical properties and creep behavior of moso bamboo. The results indicated that the density, moisture content, and flexural properties (MOR, MOE, and *ε*_u_) decreased as the heat treatment temperature was increased up to 220 °C. Additionally, heat-treated bamboo samples had higher *AWAE* and *ASE* values than the untreated samples according to the water absorption test results, indicating that samples heated at higher temperatures (>180 °C) exhibited a better water resistance. Furthermore, the master curves for all the samples using SSM with different testing parameters fit well with the 90 days of full-scale experimental data, and the construction of master curves was not influenced by the different testing parameters. The results indicated that the SSM was suitable for constructing the master curves of bamboo samples. Based on the linear slope of the Eyring plots, the activation volumes (V*) of the heat-treated bamboo samples (0.46 nm^3^ for Mo_T180_ and 0.74 nm^3^ for Mo_T220_) were lower than that of the untreated sample (0.93 nm^3^). On the other hand, the degradation ratio of the creep resistance significantly increased over all periods when the heat treatment temperature increased to 180 °C. However, the samples treated at 220 °C exhibited a reduction in the degradation ratio of the long-term creep resistance over time.

## Figures and Tables

**Table 1:** Physical and flexural properties of untreated and heat-treated bamboo samples.

Code	*ρ* (kg/m^3^)	*DLR* (%)	*MC* (%)	*MLR* (%)	Flexural Properties		
					MOR (MPa)	MOE (GPa)	*ε*_u_ (%)
Mo_NT_	721 ± 13 ^a^	–	8.9 ± 0.2 ^a^	–	135 ± 7 ^a^	10.8 ± 1.1 ^a^	2.9 ± 0.3 ^a^
Mo_T180_	682 ± 20 ^b^	4.0 ± 1.0 ^b^	6.0 ± 0.4 ^b^	33.5 ± 4.2 ^b^	133 ± 7 ^a^	11.6 ± 0.6 ^a^	1.5 ± 0.1 ^b^
Mo_T220_	642 ± 19 ^c^	10.8 ± 1.7 ^a^	5.0 ± 0.3 ^c^	44.9 ± 2.6 ^a^	91 ± 16 ^b^	10.8 ± 0.8 ^a^	1.1 ± 0.3 ^c^

Values are the mean ± SD (*n* = 8). Different letters within a column indicate significant differences (*p* < 0.05).

**Table 2:** Dimensional stability of untreated and heat-treated bamboo samples after water absorption test for 24 h.

Code	*WAR* (%)	*AWAE*	*VSC*	*ASE*
		(%)	(%)	(%)
Mo_NT_	49.3 ± 4.5 ^a^	–	10.6 ± 0.9 ^a^	–
Mo_T180_	35.2 ± 3.4 ^b^	28.3 ± 7.0 ^a^	7.9 ± 0.9 ^b^	24.1 ± 9.1 ^b^
Mo_T220_	33.2 ± 2.4 ^b^	32.4 ± 5.0 ^a^	4.5 ± 0.9 ^c^	56.3 ± 8.9 ^a^

Values are the mean ± SD (*n* = 7). Different letters within a column indicate significant differences (*p* < 0.05).

**Table 3:** Fitted creep compliances of untreated and heat-treated bamboo samples.

Code	S_0_ (1/GPa)	a	b	R^2^	*S*(*t*) (1/GPa)			*R*_d_ (%)		
					Time (Years)			Time (Years)		
					1	10	15	1	10	15
Mo_N__T_	1.51	5.86 × 10^−2^	0.38	0.9981	2.52	2.99	3.09	–	–	–
Mo_T180_	1.49	1.30 × 10^−2^	0.57	0.9855	2.42	3.14	3.32	−3.9	5.0	7.2
Mo_T220_	1.84	4.28 × 10^−2^	0.39	0.9928	2.64	3.02	3.10	4.5	0.9	0.3

*S*(*t*) = *S*_0_ + *ae^bt^*, where *S*(*t*) is the time-dependent compliance value, *S*_0_ is the instantaneous elastic compliance value, and *a* and *b* are constant values. *r*_d_ is the degradation ratio of creep resistance.

## Data Availability

Not applicable.
